# Evaluation of the Percutaneous Absorption of Drug Molecules in Zebrafish

**DOI:** 10.3390/molecules25173974

**Published:** 2020-08-31

**Authors:** Daizo Morikane, Liqing Zang, Norihiro Nishimura

**Affiliations:** 1 DIA Pharmaceutical Co., Ltd., Kashihara, Nara 634-0803, Japan; dmorikane@dia-pharma.com; 2 Graduate School of Regional Innovation Studies, Mie University, Tsu, Mie 514-8507, Japan; nishimura.norihiro@mie-u.ac.jp; 3 Mie University Zebrafish Drug Screening Center, Tsu, Mie 514-8507, Japan

**Keywords:** drug screening, felbinac, glycyrrhetinic acid, pharmacokinetics, zebrafish

## Abstract

In recent decades, zebrafish (*Danio rerio*) has become a widely used vertebrate animal model for studying development and human diseases. However, studies on skin medication using zebrafish are rare. Here, we developed a novel protocol for percutaneous absorption of molecules via the zebrafish tail skin, by applying a liquid solution directly, or using a filter paper imbibed with a chemical solution (coating). Human skin is capable of absorbing felbinac and loxoprofen sodium hydrate (LSH), but not glycyrrhetinic acid (GA) and terbinafine hydrochloride (TH). To evaluate the possibility and the quality of transdermal absorption in zebrafish, we transdermally administered these four drugs to zebrafish. Pharmacokinetics showed that felbinac was present in the blood of zebrafish subjected to all administration methods. Felbinac blood concentrations peaked at 2 h and disappeared 7 h after administration. GA was not detected following transdermal administrations, but was following exposure. LSH was not found in the circulatory system after transdermal administration, but TH was. A dose-response correlation was observed for felbinac blood concentration. These findings suggest that zebrafish are capable of absorbing drug molecules through their skin. However, the present data cannot demonstrate that zebrafish is a practical model to predict human skin absorption. Further systemic studies are needed to observe the correlations in percutaneous absorption between humans and zebrafish.

## 1. Introduction

The skin is the largest organ in the body and acts as a physical/sensory barrier to the environment, and a heterogeneous organ for drug delivery [1]. Transdermal drug delivery is a more selective and targeted delivery method in clinical practice. It can be used to continuously transport drugs through the skin, without using invasive and painful hypodermic injections, and has comparatively fewer systemic side effects compared with oral delivery [2,3]. For these reasons, topical and transdermal delivery systems of drug administration for systemic therapeutic effects are gaining attention. However, the number of drug molecules with transportability through the skin barrier that are approved by the US Food and Drug Administration is limited [4,5]. Generally, the physicochemical properties required to enable skin penetration include a molecular weight (MW) below 500 Da, and a balanced lipophilicity (log octanol-water partition coefficients (log *P*) = 1–3) [5,6]. The development of new transdermal drugs is a challenging task, as the absorption of drug molecules from the skin occurs primarily by passive diffusion [7]. As the human skin structure is composed of the epidermis, dermis, and hypodermis, drug penetration route involves (1) release from the drug formulation; (2) diffusion into the stratum corneum; (3) diffusion from the stratum corneum into epidermis layers; (4) diffusion across the epidermis layers into the dermis; and (5) absorption by the capillary network and entry into the circulatory system [8]. The assessment of transdermal absorbability of a drug involves measuring its systemic blood concentration or cumulative amount of the molecules transmitted through the skin [9,10]. In vitro and in vivo models including excised human skins, pig, rabbit, mouse, rat, and guinea pig have been developed for assessing the efficacy and the quality of topical and transdermal formulations [11,12,13]. Each animal model has its own advantages and limitations, and researchers need to choose a model that is most appropriate for the aim of the study.

Zebrafish (*Danio rerio*) is a popular and attractive vertebrate animal model for developmental and human disease studies. The characteristics that make zebrafish popular include their small size, low cost, high fecundity, optical transparency in embryo and larvae stage, rapid development and generation time, and genetic and organ similarity with humans [14,15]. Numerous human disease models of the species have been reported, including hematological disorders, muscle disorders, heart diseases, kidney disorders, cancers, obesity, and diabetes models [16,17,18]. Recently, a lecithin-based dermal delivery method was reported, by exposing the zebrafish larvae to the test reagent solution [19]. However, there is no wholly representative method for the evaluation of the percutaneous absorbability using zebrafish. Administering a drug through zebrafish skin would cause dilution and diffusion of the drug into the surrounding water, affecting the assessment of the drug. Exposing zebrafish to the chemical solution directly may result in dermal absorption; however, it could also lead to complex absorption routes simultaneously, including oral and gill absorptions.

This article aims to provide a proof-of-concept of the percutaneous absorption of zebrafish skin for the investigation of the permeation and pharmacokinetic profile of chemicals. We describe a novel protocol for the transdermal administration of drugs to adult zebrafish. To investigate the possibility of transdermal absorption in zebrafish skin, we administered felbinac, loxoprofen sodium hydrate (LSH), glycyrrhetinic acid (GA), and terbinafine hydrochloride (TH), and evaluated the blood concentration over time. We also confirmed the relationship between the administered dose and the blood concentration of felbinac.

## 2. Results

### 2.1. Leakage Test after Transdermal Administration

**Figure 1:**
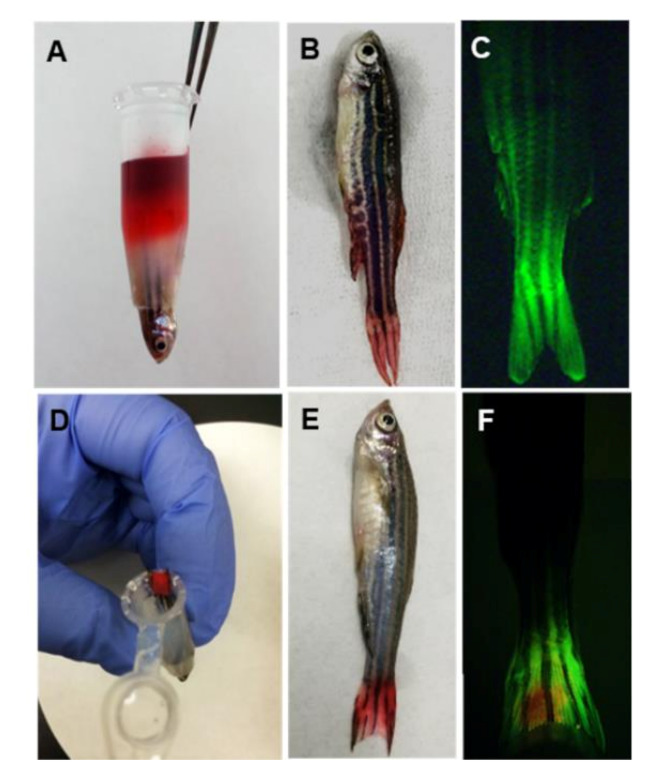
Leakage test after transdermal administration. (**A**) Transdermal administration of 100 μL of felbinac-red dye solution for 2 h; (**B**) zebrafish released from agarose gel and washed with water three times after the transdermal administration of liquid solution; (**C**) fluorescent image of the zebrafish transdermally administered with 100 μL of 4-[4-(diethylamino)styryl]-*N*-methylpyridinium iodide (4-DI-2-ASP) solution for 2 h; (**D**) the zebrafish administered with felbinac-red dye coating filter paper for 2 h; (**E**) the zebrafish released from the agarose gel and washed with water three times after transdermal administration of coating; (**F**) fluorescent image of the zebrafish transdermally administered with a 4-Di-2-ASP coating for 2 h.

We developed a novel protocol to allow drugs to be transdermally absorbed in zebrafish (Figure 1). Before performing the transdermal administration of test chemicals, zebrafish need to be fixed in a micro-centrifuge tube filled with low-melting-point agarose gel, except for the head/gill and the tail. Agarose gel is a three-dimensional matrix with channels and pores that allow biomolecules to pass. To demonstrate that our protocol achieved transdermal administration with no oral and gill absorption, we performed a leakage test using the liquid solution and coating methods. Firstly, we transdermally administered 100 μL of felbinac-red dye solution, which caused the drug to disperse down toward the head of the zebrafish after 2 h; however, the front line of the red dye did not reach the end of the agarose gel (Figure 1A). The zebrafish released from the agarose gel revealed that the red dye just reached the middle of the body, and no dye dispersed to the gill or the mouth (Figure 1B). We collected the anesthetic solution at 0, 1, and 2 h after the administration, to determine the concentration of felbinac using HPLC, and measured the absorbance at 510 nm to detect the red dye. Neither the felbinac nor the red dye was detected, which means that no test chemicals leaked through the agarose gel. Similarly, transdermal administration with 100 μL of 4-Di-2-ASP solution for 2 h showed that the fluorescent signals were mainly observed in the tail fin and part of the posterior body, with no signals found in the anterior body (Figure 1C).

For the leakage test of coating transdermal administration, the zebrafish were administered the felbinac-red dye solution for 2 h (Figure 1D). Visual observation showed that only the tail fin was stained with red color (Figure 1E). The anesthetic solutions were collected and measured as described above, and no felbinac or red dye was detected. Furthermore, the fluorescent signal was limited to the tail fin (Figure 1F).

### 2.2. Pharmacokinetic Analysis of Test Drug Molecules in Zebrafish

**Figure 2:**
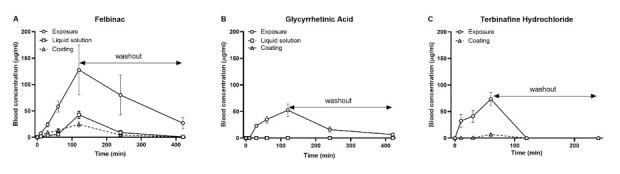
Mean blood concentration-time profiles of felbinac (**A**), glycyrrhetinic acid (GA) (**B**), and terbinafine hydrochloride (TH) (**C**) after exposure and transdermal administrations. Each point and vertical bar indicate the mean and SD (standard deviation), respectively. *n* = 5.

Felbinac was transdermally administered to zebrafish using the liquid solution and coating methods in equivalent amounts. The blood concentrations of felbinac over time were measured by HPLC analysis. No interfering peak was detected from the chromatogram of the blank sample (untreated control) at the retention time that corresponds to felbinac (data not shown). Figure 2A shows the time-dependent change in the average concentration of felbinac in blood plasma collected from zebrafish after exposure administration or liquid solution and coating transdermal administration. For all the three methods, the maximum blood concentration values of felbinac were detected at 120 min after administration, just before washout. These results demonstrated the continuous absorption of felbinac via the skin, the gill, and the mouth (exposure administration) or via the skin only (liquid solution and coating transdermal administration). The observed maximum blood concentrations of felbinac were 127.8 ± 47.3, 42.5 ± 6.59, and 24.46 ± 4.51 μg/mL after exposure, liquid solution, and coating administration, respectively. The areas under the blood concentration-time curve corresponding to the above were 508 ± 199, 93.3 ± 23.4, and 74.9 ± 35.1 μg h/mL, respectively. After the washout of felbinac at 120 min, the blood concentrations of felbinac after exposure administration decreased with a half-life of approximately 2 h (80.1 ± 37.6 μg/mL). The drug was not completely metabolized after 5 h (26.9 ± 10.7 μg/mL). Additionally, the blood concentrations of felbinac after liquid solution and coating transdermal administration decreased rapidly after a 2 h washout (8.87 ± 3.86 μg/mL and 4.2 ± 2.15 μg/mL, respectively), with the drug being almost completely metabolized after 5 h (0.81 ± 0.69 μg/mL and 0.69 ± 0.61 μg/mL, respectively). Additionally, LSH, a topical and transdermal drug used in humans, was not detected in the plasma of zebrafish after exposure and transdermal administration. To clarify if these results were caused by the low dose of LSH, we challenged a 10-fold dose, 10 μg/mL of exposure and 304 μg of coating transdermal administration for 30 min, but still failed to detect this drug in the circulatory system of zebrafish.

We measured the blood concentrations of GA and TH as negative controls, as they cannot be absorbed by human skin. The exposure administration of GA showed a time-dependent increase in blood concentration, which decreased after the drug washout (Figure 2B). Consistent with the phenomenon observed in humans, no GA was detected in the circulatory system of zebrafish, by either the liquid solution or coating transdermal administration. However, TH was detected after coating transdermal administration (Figure 2C). Different from the other three drugs, the equivalent amount of TH treatment caused high mortality after 2 h of administration. Therefore, we shortened the administration time to 60 min and found that the maximum blood concentrations of TH were 70 ± 10.1 and 7.5 ± 1.3 μg/mL after exposure and coating transdermal administration, respectively. The area under the blood concentration-time curves corresponding to the above were 79.7 ± 12 and 5.5 ± 1.1 μg h/mL, respectively. After the washout, the blood concentrations of TH decreased quickly and TH was completely metabolized within 1 h, either after exposure or after transdermal administration.

### 2.3. Dose-Related Blood Concentration of Felbinac by Transdermal Administration

**Figure 3:**
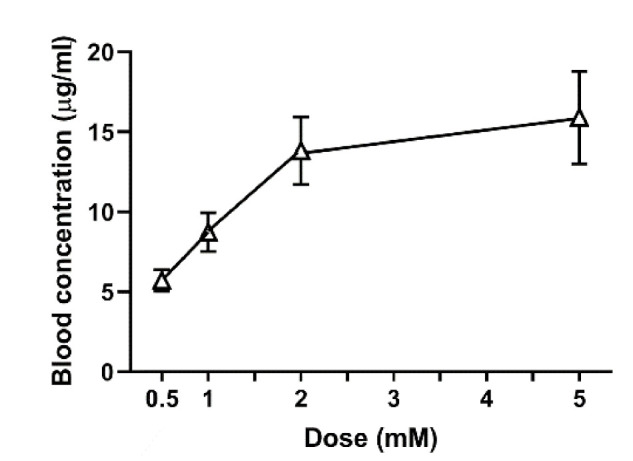
Blood concentrations of felbinac with increasing dose after coating transdermal administration. Each point and vertical bar indicate the mean and SD, respectively. *n* = 5.

To investigate if the transdermally applied amount of felbinac could affect its blood concentration, we performed a dose-response analysis (Figure 3). Using coating transdermal administration protocol, we applied 5 μL of 0.5, 1, 2, and 5 mM felbinac to the zebrafish tail (containing 5.3, 10.6, 21.2, and 42.4 μg felbinac, respectively). The blood concentrations of felbinac showed an increase in a dose-dependent manner (5.47 ± 0.19, 8.62 ± 1.84, 13.70 ± 3.06, and 16.10 ± 5.81 μg/mL, respectively).

## 3. Discussion

Transdermal absorption is one of the common drug administration methods in humans. However, there are no established guidelines for transdermal administration using the zebrafish, despite being a widely used model organism. In this study, we developed a novel protocol that achieved transdermal administration uncontaminated with the other absorption routes using adult zebrafish. Using this technique, we found that zebrafish skin can absorb drugs transdermally in a dose-dependent manner, and the absorbability varies depending on the physicochemical properties of the chemical substance.

We developed two methods to administer the test drug molecules, including liquid solution and coating transdermal administration. Although zebrafish were fixed in an upside-down position under anesthesia, there was a possibility of chemical leakage by dispersion through the agarose pores. To exclude this possibility, we investigated the chemical leakage using the two methods. Both liquid and coating transdermal administration approaches did not produce any leakage after 2 h of treatment using red dye or fluorescent dye (Figure 1). The anesthesia solution also did not detect any target chemicals. These results indicate that our protocol achieved pure transdermal administration with no oral or gill absorption. For the individual difference of the sensitivity to the anesthesia, we recommend confirming chemical leakage, to ensure transdermal absorption by examining a suitable application time and measuring the amount of test chemicals in the anesthesia solution at the end of the test. To decrease the leakage risk, we suggest performing the liquid solution administration within 2 h. As the absorption area is an important factor that affects the stability of the test results, we recommend choosing zebrafish individuals with similar body length and body weight.

Between the two transdermal administration methods, researchers can select the right one based on the purpose and the characteristics of the test chemical. Liquid solution transdermal administration is a simple approach for the percutaneous absorption screening of chemicals. It is also suited for assessing the short-term treatment of large amounts of the test molecule. However, as agarose used to fix the zebrafish can leak chemicals through passive diffusion during long-term treatment, the application area for the test chemical may spread over time and affect the speed and the quantity of absorption. Therefore, it is necessary to examine the application time according to the purpose of the test and the properties of the chemical substance. The advantages of the coating transdermal administration include the low possibility of test substance leakage and the ability to minimize differences between experimental systems, in terms of the quantity and the speed of transdermal absorption. However, the test substance is limited in terms of the amount that can be dissolved in small quantities of a solvent (in this study, 5 μL). Additionally, when applying the coating administration, it is important to (1) choose a suitable solvent to ensure that the substance can be dissolved (at least part of the substance needs to be dissolved) and (2) choose a solvent with low volatility at room temperature, to ensure that it remains closely affixed to the site of application. We will consider the application of adhesive agents, ointments, or gel-like drugs instead of filter paper directly to the zebrafish skin in future studies using this protocol.

Felbinac is a potent nonsteroidal anti-inflammatory drug that is widely used for the treatment of osteoarthritis, muscle inflammation, and acute soft tissue injuries [20,21,22]. Owing to its adverse effects concerning the gastrointestinal tract, the most common formulation for administering felbinac is the topical formulation for transdermal drug delivery. It can be detected in the blood after skin penetration [23]. Mainly administered orally in humans, GA is an active aglycone of glycyrrhizin, which possesses anti-inflammatory, hepatoprotective, antitumor, and anti-viral pharmacological activities [24,25,26,27]. For skin penetration, the physicochemical properties of molecular weight <500 Da and log *P* = 1–3 are required; felbinac satisfies these conditions, but GA does not. Although GA is also used in cosmetics, its log *P* is too high to enable penetration through the human skin. Therefore, strategies to enhance skin penetration have been developed [28], such as the use of an organic base and non-aqueous solvent system [29], an elastic liposomal system [30], or a nanoemulsion system [31]. In our study, we did not use these systems to enhance the transdermal delivery of GA; therefore, we hypothesized that the transdermal treatment of GA would not occur in zebrafish. In the application of our novel transdermal administration protocol, we analyzed the pharmacokinetics of felbinac and GA via transdermal administration (Figure 2). Felbinac was transdermally absorbed in zebrafish but GA was not, which was consistent with our hypothesis. Although GA was detected in the blood through exposure administration, it was absorbed by the digestive system and the gills and not through the skin of zebrafish. We also observed that the blood concentration of absorbed felbinac increased with time and disappeared over time upon discontinuation of administration. Liquid solution and coating administrations showed different trends of felbinac blood concentrations, despite using the same dose. These differences may be explained by the difference in the quantity of the felbinac absorbed, owing to the expansion of the application area during the liquid solution administration method. This result suggested that the application area would affect the quantity of the transdermally absorbed test molecule. To avoid the effect of application area change caused by liquid solution transdermal administration, we developed the coating filter paper-based topical application. Transdermal administration of felbinac resulted in rapid penetration through the skin to the circulation in a dose-dependent manner (Figure 3), which was similar to the results observed in rats [22].

We obtained results that are inconsistent with those observed in human skin regarding LSH and TH. LSH is a nonsteroidal anti-inflammatory drug that can be absorbed by human skin [32]. However, we did not detect it in the blood of zebrafish after exposure and transdermal administrations, even with a high dose. We supposed that percutaneous absorption had happened, but rapid metabolism occurred when LSH entered the circulation because it is a prodrug of the pharmacologically active trans-OH form [33]. TH is an orally and topically active allylamine antifungal agent [34]. Although TH is used as a topical drug, it cannot transdermally penetrate the stratum corneum of human skin, because it has a high binding affinity (96%) to the major protein in the stratum corneum, keratin [35]. However, in our study, TH was detected in the circulatory system of zebrafish after transdermal administration. This result may be caused by the special skin characteristics of zebrafish. Although the skin of zebrafish also contains an epidermis, a dermis, and a hypodermis, similar to humans, there is no stratum corneum [36]. We believe that the drug penetration route in zebrafish skin follows this process: (1) release from the drug formulation; (2) diffusion into the epidermis layers; (3) diffusion into the dermal layers; and (4) absorption by the blood vessels and entry into the circulatory system. Another issue that cannot be ignored is the thickness of the zebrafish skin layer. Compared with the skin thickness of ~3 mm in adult humans [37], the average full-skin thickness in a 1-month-old zebrafish is only 14 µm [36]. Consequently, the lack of the stratum corneum (=shortened drug penetration procedure) and the very thin skin of zebrafish leads to different chemical absorption rates compared with mammals. For this reason, although we successfully demonstrated the transdermal absorbability of zebrafish skin, further research is required to compare the results obtained among species. Numerous molecules with different molecular weights and different partition coefficients need to be tested to identify the relevance of zebrafish as a practical model of percutaneous penetration in humans. Our study provided a novel experimental method, which can be considered as the first step to assess transdermal drugs using zebrafish.

## 4. Materials and Methods

### 4.1. Ethics Statement

All animal procedures were performed according to Japan’s Act on Welfare and Management of Animals (Ministry of Environment of Japan), and complied with international guidelines. Ethical approval from the local Institutional Animal Care and Use Committee was not sought, as this law does not mandate the protection of fish.

### 4.2. Zebrafish Strain and Their Maintenance

AB strains (Zebrafish International Research Center, Eugene, OR, USA) were maintained at 28 °C, with a light/dark cycle of 14/10 h [38]. The adult zebrafish used in this study were 4–6-month-old males. The fish were fed GEMMA Micro 300 (Skretting, Stavanger, Norway) daily. Additionally, the fish were fed live *Artemia nauplii* once a day.

### 4.3. Chemicals

Felbinac, also known as Biphenyl-4-ylacetic acid (MW = 212.24 Da, log *P* = 3), was purchased from Xunda Pharmaceutical Co., Ltd. (Wuxue, Hubei, China). The molecular weight of GA (Alps Pharmaceutical Ind. Co. Ltd. Hida, Gifu, Japan) is 470.68 Da, and the log *P* is 6.4. LSH (MW = 304.31 Da, log *P* = 0.82) and TH (MW = 327.89 Da, Log *P* = 7.4) were obtained from Hamari Chemicals (Osaka, Japan). The fluorescent dye 4-[4-(diethylamino)styryl]-*N*-methylpyridinium iodide (4-Di-2-ASP) was purchased from Sigma Aldrich (St. Louis, MO, USA). Red dye No. 102 (Ponceau 4R) was purchased from Fujifilm Wako (Osaka, Japan). All the chemicals were dissolved in ethanol to prepare a 1 mM working solution.

### 4.4. Transdermal Administration

#### 4.4.1. Preparation

**Figure 4:**
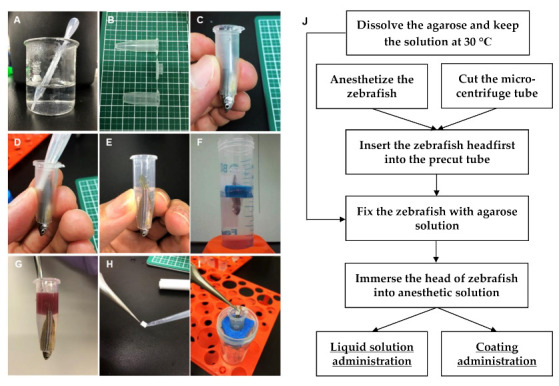
Procedure for transdermal administration in adult zebrafish. (**A**) Dissolved 3% agarose solution; (**B**) A 1.5 mL micro-centrifuge tube with the tip cut off up until approximately 10 mm; (**C**) anesthetized zebrafish inserted into the precut micro-centrifuge tube; (**D**) zebrafish fixed with agarose solution; (**E**) zebrafish fixed with hardening agarose; (**F**) fixed zebrafish immersed into anesthetic solution; (**G**) liquid solution applied into the fixing tube; (**H**) filter paper cut to approximately 2.5 × 5-mm pieces, and the test chemical (coating) applied; (**I**) filter paper affixed to the tail fin using forceps; (**J**) schematic of the transdermal administration procedure.

Agarose (A5093; Sigma Aldrich) and low-melting-point agarose (Agarose L; NIPPON GENE CO., LTD., Tokyo, Japan) were mixed at a 1:1 ratio and added to water to produce a 3% aqueous solution. This solution was brought to a gentle boil in a glass beaker to solubilize agarose. When the solution was clear and colorless (Figure 4A), it was moved to a water bath or a hot plate, and allowed to cool to 30 °C, and then kept at this temperature.

Adult zebrafish were anesthetized by placing them in a tank containing 0.168 mg/mL of Tricaine (Fujifilm Wako) solution for 15–30 s. The tip of a 1.5 mL micro-centrifuge tube was cut to 5–10 mm size using fine scissors or a cutter (Figure 4B). The size of the hole was dependent on the size of the zebrafish head. Using a skimmer spoon, the anesthetized fish was then lifted from the Tricaine bath and gently slipped headfirst into the tip-cut micro-centrifuge tube (Figure 4C). It was necessary to leave the gills of the zebrafish out of the hole to ensure breathing, while taking care not to let the zebrafish slip through the hole. If the hole size of the tube was too narrow, the fish was put back into the anesthetic solution and gently pushed out the tube. The tip was cut again to make minor adjustments. Next, zebrafish were fixed by quickly pouring the agarose solution into the tube up to the base of the tail fin (Figure 4D,E). The fixed zebrafish with exposed tail fins were inserted to the floater, and their ability to breathe was secured by immersing their head in a water tank containing 30–50 mL of 0.06 mg/mL Tricaine solution (Figure 4F). After several minutes, the agarose hardened, and the zebrafish for transdermal administration was ready for drug administration. Fish were placed at room temperature (25~28 °C), or in an incubator at 28 °C. The vital signs were checked occasionally by observing gill and mouth movements.

#### 4.4.2. Liquid Solution Transdermal Administration

The test chemical solution (100–500 μL) was directly applied to the fixing tube until the tail was completely immersed (Figure 4G). The fixed zebrafish were kept submerged in the anesthetic solution for a maximum of 3 h. After administration, the chemical solution was removed, and the zebrafish were gently released from the agarose gel. The zebrafish were washed three times with 500 mL of water before sampling for blood collection.

#### 4.4.3. Coating Transdermal Administration

The filter paper was cut to 2.5 × 5 mm pieces, after which five microliters of test chemical solution was applied (Figure 4H), and then the paper was affixed directly to the tail fin of the zebrafish (Figure 1I). After the end of the test, the filter paper was removed, and the zebrafish were gently removed from the fixing tube. The zebrafish were then washed three times with 500 mL of water to provide a sample for blood collection.

### 4.5. Transdermal Administration of Test Drugs

Adult zebrafish with similar body length and body weight were randomly divided into several groups, each containing five fish. In the test for the liquid solution transdermal administration, 100 μL of 1 mM test drugs working solution was administered to the anesthetized zebrafish. For the coating administration test, a piece of paper was impregnated with 5 μL of a solution, in which 21.2 μg of felbinac, 47 μg of GA, 30.4 μg of LSH, or 32.8 μg of TH was dissolved (water: propylene glycol (Fujifilm Wako): ethanol = 1:1:1) and affixed to the tail fin of the zebrafish. The administration period was 60 min for TH and 120 min for the other three drugs, and then the chemicals were washed out. At 10, 30, 60, and 120 min after the initial administration, as well as 120 min and 300 min (60 min and 180 min for TH) after the test molecules washout, 5 μL of blood was collected as previously reported [39,40]. Ethanol was added to the blood samples to adjust the final volume to 250 μL, and then vortexed and centrifuged at 7700× *g* for 10 min. The supernatants were then collected for analysis using high-performance liquid chromatography (HPLC).

### 4.6. Exposure Experiment

Adult zebrafish were exposed to 1 μg/mL of test drug solution, then blood samples were taken at the same conditions of the transdermal administration test, and the blood concentrations of test drugs were measured using HPLC analysis.

### 4.7. HPLC Analysis

Test drugs were dissolved in ethanol to produce a 5 mg/mL stock solution for calibration standard preparation. The stock solution was diluted with ethanol to produce 0.15, 0.45, 0.75, 1.00, and 1.50 μg/mL of calibration standards. HPLC was performed as previously reported [41]. In brief, a 20 μL aliquot of sample supernatant was analyzed by an HPLC system consisting of an L-7100 pump, an L-7200 autosampler, an L-7300 column oven, an L-7400 UV detector (Hitachi High-Technologies, Tokyo, Japan), and a Shimadzu CR-7A computing integrator (Shimadzu, Kyoto, Japan). The system was equipped with a 4.6 × 250 mm C18 column (6.5 μm particle size, Fujifilm Wako) maintained at 40 °C. Elution was carried out isocratically with a mobile phase consisting of acetonitrile: phosphoric acid (0.01 M; 1:1, *v*/*v*) at a flow rate of 1 mL/min. The eluate was monitored at a wavelength of 254 nm. Blank data were obtained from the blood of the control fish, as described above. Drug concentrations were determined by measuring the peak area and comparing it with the peak area of known standards. Interventional studies involving animals or humans and other studies require ethical approval, and must list the authority that provided approval and the corresponding ethical approval code.

### 4.8. Leakage Test for Transdermal Administration

Transdermal administration of felbinac was conducted through the tail fin of zebrafish. This was performed using (1) 100 μL of felbinac-red dye solution containing 21.2 μg felbinac and 75 μg red dye and (2) a filter paper impregnated with 5 μL of felbinac-red dye solution (water: propylene glycol: ethanol = 1:1:1 solution containing 21.2 μg felbinac and 75 μg red dye). One milliliter of 0.06 mg/mL Tricaine solution used to secure the fish’ breathing was sampled at 0, 1, and 2 h after the start of the test. The concentration of felbinac in these sampled anesthetic solutions was quantified using HPLC, as described above. To detect red dye, the absorbance of the sample solution was also measured at 510 nm, using a spectrophotometer (Hitachi U-3300; Hitachi, Tokyo, Japan). Furthermore, red dye deposits in the zebrafish were visually observed after the test.

In the leakage test using a fluorescent dye, 100 μL of 1 mM 4-Di-2-ASP was administered to zebrafish through the tail fin for 2 h, and a filter paper containing 39 μg of 4-Di-2-ASP dissolved in 5 μL of ethanol was affixed directly onto the tail fin of the zebrafish for 2 h. After administration, the zebrafish were washed with water three times. The fluorescent signal in the zebrafish body was observed via images captured using an Olympus SZX7 microscope with a GFP filter (Olympus, Tokyo, Japan).

### 4.9. Dose-Response Study of Felbinac by Transdermal Administration

Solutions were prepared to contain 5.3, 10.6, 21.2, and 42.4 μg of felbinac in 5 μL of solution. Each of these 5 μL solutions was applied to filter paper adjusted for coating administration purposes, then affixed to the tail fin of the zebrafish for 1 h. After administration, the zebrafish were washed with water three times, and blood samples were collected for measurement of felbinac blood concentration using HPLC. The quantity of felbinac in the anesthetic solution was also determined.

### 4.10. Statistical Analysis

Results are reported as means and standard deviations. Statistical analyses were performed using the Student’s *t*-test or one-way analysis of variance, followed by Dunnett’s multiple comparison test, using GraphPad Prism version 8 (GraphPad Software Inc., San Diego, CA, USA). A value of *p* < 0.05 was considered statistically significant.

## 5. Conclusions

We developed a novel protocol for transdermal administration using zebrafish, and provided a proof-of-concept that zebrafish can absorb drug molecules through their skin. We evaluated percutaneous absorption based on molecular weight and partition coefficient; however, it was found to be affected by factors such as drug formulation or differences in moisture in the skin. Based on the limitation that the structure of zebrafish skin is different to that of human skin, further systemic studies are needed to observe the correlation in percutaneous absorption between humans and zebrafish.
